# Bibliographical Notices

**Published:** 1844-01-01

**Authors:** 


### Periscope;

OR,

### CIRCUMSPECTIVE REVIEW.

"Ore trahit quodcunque potest, atque addit acervo."

### Notices of some New Works.

### An Essay on Thunder-Storms—their Phenomena, and the Means of Safety from their Effects. By *E. A. Turley*, M.D., &c.

The neighbourhood of Worcester having been visited by an awful thunder-storm during the Summer of 1843, our author, finding great misapprehension prevailing among the public from these terrific phenomena, was induced to write a small Essay on the identity of electricity and lightning, with a statement of some of its known laws. The public are not aware that fifty thousand pounds' worth of property is annually destroyed by this gigantic element, and that, since the year 1793, more than 250 ships of war have suffered by thunder-storms—70 seamen have been killed—and 130 wounded. It is not in our power to follow our author through the details of his Essay, but to exhibit a sample or two that will, at once, prove interesting in themselves, and shew the ability with which our author manages his subject.

"A thunder-storm rarely arises without a previous sunny day, during which the vapour of the earth has carried with it a portion of positive electricity; for it must be borne in mind that water changed to vapour elicits electricity. The vapour being lighter than the air, ascends till it meets with a rarer atmosphere, and is carried by the wind away from the surface of the earth, whence it arose. There it perhaps meets with a colder medium, becomes condensed, and descends very near to the earth, till at last it is averted in its course by the attraction of the earth oppositely charged—that is, with negative electricity. There is now set up an attraction betwixt the earth and the cloud, and it requires only some conductor—such as a tree or tall house, a chimney or a high hill, to form a medium of union, when a discharge is made, and the equilibrium is restored. We observe the flash, and call it lightning; but if we had pushed together two vessels charged as the clouds were, the one with positive and the other with negative electricity, till they nearly touched, we should then have seen a flash, which we call the electric spark: we hear a snap at the same instant, which in the clouds we should have called thunder. Hence lightning is the electric flash, and the displacement and consumption of a portion of the air causes the thunder. The sulphureous smell which often accompanies the atmospheric discharges is produced by a decomposition of a portion of the atmospheric air by the lightning, the result being the evolution of nitrogen gas. It may prove interesting to the reader to direct his attention to what visibly takes place in the appearance of the clouds during a thunder-storm, and these appearances were well marked on Wednesday, August 9th. From an elevation in St. John's in the morning of that day the thunder cloud was seen in the direction N.N.W.; on each side of it and above it were light clouds, with jagged edges, moving in opposite and variable directions, as though impelled by different currents. The atmosphere was calm and warm, producing a feeling of faintness and oppression; the cattle were moving about uneasily, but not grazing. A discharge took place in the direction of Hallow, when immediately all the lighter clouds hastened to the thunder cloud, as though they were anxious to contribute their quota of fresh ammunition. At one period there were four currents moving the light clouds in alternate layers in opposite directions. The rain now fell in torrents, mixed with a little hail. The black cloud wheeled on towards Barbourne, when an almost perpendicular flash of a yellow colour and zig-zag form, again discharged the cloud, which now took a circuit towards Broadway. Four times did Worcester appear the centre of the electric circuit in the course of the day. The thunder was heard at Worcester, more or less, during nineteen hours, viz., from three a.m. till ten p.m."

MEANS OF SAFETY FOR MAN AND ANIMALS.

"Supposing, then, a person overtaken by a thunder-storm on a hill or mountain—what should he do? let him immediately descend, or descend in part, and lie down. If he be in a field? let him lie flat down, or betake himself to a hedge, near to a tree, if possible, and yet not within twenty yards of it. If on horseback? let him dismount and turn his horse adrift. If in a carriage? close it, and sit down in the middle of it. In a barn? let him avoid the walls. Should he be in a vessel at sea? let him avoid the masts and forecastle, and betake himself to his hammock, except he be on board an iron vessel, and then he will be perfectly protected. In a boat? furl the sails, rear a wet oar near the head of the boat, and retire to the stern and lie down. If in a house? let him, if possible, choose a middle chamber, place the bed in the middle of the room through which, if possible, no flue passes, and there he may lie down in tolerable security. If a female is alarmed on seeing the lightning? let her take her seat in the cellar, far away from the walls, and especially from the foundation of a chimney. A cellar affords good security against the upward or negative current, whilst a bed affords the best security against the effects of the downward bolt or positive current. A position near a lake or stream of water is highly dangerous. The centre of a *railway carriage*, at a distance from the engine, is perhaps the securest place of all. The rails dissipate the negative charge; the low flat on which they are placed renders them safer than higher uneven ground; and should a train be struck, the chimney and engine would be the parts most attractive. As yet we have not heard of an accident on any line from lightning. The Glasgow and Liverpool mail was struck in 1836, when three outside passengers were killed, while those inside escaped uninjured. When lightning once enters a room, it will leave traces of its powers on almost everything of a metallic nature near to the chimney. Lightning rarely passes an open space, unless conducted to it by metal. Windows are often broken by the concussion of the air, even at a considerable distance from the tract of the lightning. Since a current of moist air may become a conductor, a position betwixt an open door and window should be avoided. Some persons *foolishly open their doors and windows during tempests;* this practice admits a current of damp air, which becomes a conductor of electricity; as is exemplified by the difficulty of performing electrical experiments in moist or cloudy weather. A pocket electrometer might be constructed for a trifle, which would warn the possessor of his danger. People will often assert that they have seen the lightning playing on the bell-wires and other substances; the absurdity of such statements is best shewn by reference to the fact that lightning travels at the rate of 576,000 miles in a second of time, and that it could be conveyed from this city to London and back in the twinkling of an eye; in short, in no appreciable time." 21.

The whole of the Essay is written with equal talent and utility, as is exhibited in the foregoing extracts.

Elementary Instruction in Chemical Analysis. By Dr. *C. R. Fresenius*. With a Preface by Professor *Liebig*. Edited by *J. Lloyd Bullock*. Octavo, pp. 284. Churchill.

It cannot be questioned that medicine is destined shortly to undergo great changes from the advance of chemistry. The methods devised within a few past years for investigating the constitution of organic bodies, and for tracing the changes they pass through under the influence of the vital principle, have brought this hitherto mysterious part of nature within the reach of inductive science. Their application to the products of disease must now follow, and the agency of chemical forces in originating or sustaining diseased action be determined. Whether the sanguine expectations of the chemico-pathologists be realized to their full extent or not, here is an unexplored field for investigation, and treasures of new truths and principles lie beneath the surface to reward the enquirer. We would strongly recommend our junior brethren to turn their attention this way, and to employ their leisure in making themselves practically expert in chemical processes. Of course a complete knowledge of inorganic chemistry is a preliminary qualification for the branch more immediately belonging to the physician, but this may be obtained with so much ease as to deserve only the name of a recreation. The work before us is eminently calculated to afford facilities for this object, and with respect to its character, we cannot say more than has been said by an able critic, "Liebig."

"Dr. Fresenius conducts the course of elementary instruction, in mineral analysis, in the laboratory of the University of Giessen. During the two last sessions he has followed the method described in his work, entitled, 'Elementary Instruction in Qualitative Chemical Analysis.' This method I can confidently recommend from my own personal experience to all who are desirous of obtaining instruction in inorganic analysis, for its simplicity, usefulness, and the facility with which it may be apprehended.

"I consider Dr. Fresenius' work extremely useful as an introduction to Professor H. Rose's excellent manual, and for adoption in institutions where practical chemistry is taught, but it is especially adapted to the use of Pharmaceutical Chemists.

"Further, a number of experiments and discoveries have been recently made in our laboratory, which have enabled Dr. Fresenius to give many new and simplified methods of separating substances, which will render his work equally welcome to those who already are familiar with the larger works on inorganic analysis.—*Justus Liebig*."

After this opinion of Dr. Fresenius' work we should think it quite indispensable to the chemist, and to every medical student who wishes to obtain any skill in practice, or whose views extend beyond the small amount of chemistry to be learned from lectures. He will find this work the simplest and most lucid guide for the operations of the laboratory existing, and we congratulate the English student upon its appearance in our language.

---

Blood and Urine of Chlorotic Subjects. (Auszug aus Buchner's Repertor., II. Reihe, Bd. 29, Heft 2.)

The results which have been obtained from the experiments instituted by Andral, Gavarret, and Simon, on the blood of chlorotic patients, before and after the use of ferruginous medicines, and which coincide so closely, find an additional confirmation in the experiments recently instituted by Herberger. Of the preparations of iron, those employed were the ferrum alcoholisatum, Tinctura ferri acetici ætherea, and the Vinum ferri—their use was continued for the space of eight weeks. The patient was a young girl about 20 years of age, who had repeatedly tried the Schwalbach bath without any permanent benefit. The following analyses show the composition of the blood before the use of the iron (A), and after the use of the iron (B). The blood, after spontaneous coagulation, formed a somewhat copious coagulum without the inflammatory coat.

| | A. | B. |
|---|---|---|
| Water .. .. .. .. .. .. .. .. .. | 868,34 | 807,08 |
| Solid constituents .. .. .. .. .. .. | 131,66 | 192,92 |
| Fibrin .. .. .. .. .. .. .. .. .. | 3,609 | 1,950 |
| Albumen .. .. .. .. .. .. .. .. .. | 78,200 | 81,509 |
| Globulin .. .. .. .. .. .. .. .. .. | 36,470 | 94,290 |
| Hæmatin .. .. .. .. .. .. .. .. .. | 1,590 | 4,029 |
| Fat .. .. .. .. .. .. .. .. .. .. | 2,310 | 2,470 |
| Extractive matter and salts .. .. .. .. | 8,921 | 8,236 |
| Loss .. .. .. .. .. .. .. .. .. .. | 0,500 | 0,409 |

These experiments approximate very closely to those instituted by Simon;* he too observed that, notwithstanding the extraordinary increase of Hæmatoglobulin after the use of iron, the fibrin had diminished, and that, before as well as after the treatment, the quantity of albumen continued the same.

Herberger's experiments are particularly interesting for this reason, that he at the same time examined the urine of the patient before and after the use of the iron; from Becquerell's observations (Semeiotique des urines, p. 291) it may be seen that, simultaneously with the diminution of the blood-corpuscles, the quantity of the urea in the urine diminishes, so that in the one case the quantity of the urea in the urine of chlorotic subjects scarcely amounts to more than the half of that contained in normal urine. Nearly the same proportions result from Herberger's experiments; the urine of a chlorotic girl was examined on three different days during her illness, and on two different days after her recovery.

*Urine before the Use of Iron.*

| | 1 | 2 | 3 |
|---|---|---|---|
| Spec. grav. .. .. .. .. | 1,010 | 1,009 | 1,012 |
| Quantity of urine passed in 24 hours | 32oz. | 42oz. | 35oz. |
| Water .. .. .. .. .. | 975,43 | 978,21 | 971,98 |
| Solid matters .. .. .. .. | 24,57 | 21,79 | 28,02 |
| Urea .. .. .. .. .. | 7,04 | 7,00 | 7,12 |
| Uric acid .. .. .. .. | 0,13 | 0,21 | 0,19 |
| Extractive matter .. .. .. | 10,48 | 9,00 | 13,99 |
| Fixed salts .. .. .. .. | 6,80 | 5,50 | 6,62 |
| Loss .. .. .. .. .. | 0,12 | 0,08 | 0,10 |

*Urine after the Use of Iron.*

| | 1 | 2 |
|---|---|---|
| Water .. .. .. .. .. | 940,16 | 938,70 |
| Solid matters .. .. .. .. | 59,84 | 61,30 |
| Urea .. .. .. .. .. | 26,84 | 27,36 |
| Uric acid .. .. .. .. .. | 0,94 | 0,96 |

* Medicinische Chemie, Bd. II, S. 207.

| | 1 | 2 |
|---|---|---|
| Extractive matter.. .. .. .. | 18,62 | 16,28 |
| Fixed salts .. .. .. .. | 13,32 | 15,71 |
| Loss .. .. .. .. .. | 0,12 | 0,99 |

If the quantity of the urea and uric acid per cent. in the solid residue be calculated from these analyses, the following numbers are found to result in the same series as above.

| | 1 | 2 | 3 | 4 | 5 |
|---|---|---|---|---|---|
| Urea .. .. .. | 28,7 | 32,1 | 25,4 | 44,9 | 46,2 |
| Uric Acid .. .. .. | 0,5 | 0,9 | 0,7 | 1,5 | 1,5 |

The striking difference in the quantities of the urea and uric acid, which the urine contains before and after the use of iron, appears from hence sufficiently manifest. In order to form a correct estimate, however, reference must be had to Lehmann's observations respecting the influence of diet on the urea and uric acid contained in the urine. Herberger remarks that the girl, after she recovered, indulged her appetite very much with a plentiful quantity of animal food. On the other hand, it must not be overlooked that, in the case of chlorotic individuals, nitrogenized articles of food are not excluded; in this instance, therefore, there may be merely an increase in the quantity of the ordinary food, and so the great increase in the quantity of the urea may be in part referrible to the changed composition of the blood. Herberger satisfied himself that the urine during the process of cure contained iron, more especially in the morning, and also that the perspiration contained the same substance.—*(Beiträge zur Physiologischen und Pathologischen Chemie und Mikroscopie von Dr. Fr. Simon, Berlin,* 1843.)

### Contributions to the Subject of the Action of Medicines.

### *On the Action of Alcohol and Ether on the Animal System.*

C. H. Mitscherlich has instituted several experiments. That alcohol passes into the blood, that it is voided partly through the pulmonary transpiration, partly through the urine, follows from the experiments of Orfila, Magendie, Percy, and several others. Anhydrous alcohol coagulates the soluble proteine combinations, as albumen and caseine, and changes them into those modifications that are insoluble in water; in the same manner the albumen which is mixed with the contents of the stomach is coagulated; alcohol applied to the external skin first produces a feeling of cold by evaporation, sometime after, on the contrary, heat and burning are felt. On account of the indurated and dried state of the cells which constitute the epidermis, the latter takes in the alcohol slowly; on the contrary, the alcohol penetrates the epithelium much easier; it soon excites a feeling of burning, which is again followed by inflammation, the epithelia change their form, and shrink; in recent wounds, in which the nerves are exposed, alcohol produces the most acute pain, and soon after inflammation. Rabbits, into whose stomachs one ounce of anhydrous alcohol had been injected, fell in a short time into a state of great faintness, sensation and motion were diminished, so that the animals could no longer keep themselves upright, the pulse and respiration were very much hurried, death followed from one hour and a half to two hours without any convulsions; on instituting an examination immediately after death, the alcoholic odour was palpable, the muscles still contracted when mechanically irritated, the peristaltic motion of the intestinal canal was weak, the stomach was found to be very much inflamed; the colour of the small intestine was not changed; the gastric contents smelt strongly of alcohol, and were partly coagulated. The chemical action of alcohol on the coats of the stomach was placed beyond all doubt; the epithelium was detached only in some places, it was of a grey-white colour, and easily torn, the form of its cells was changed, they appeared, as it were, shrivelled up. The vascular membrane was a dark-red brown, full of blood, and penetrated with a partly clear or reddish-coloured exudation, whereby it was increased considerably in thickness, but the exudation itself was not coagulated; the muscular coat and the peritoneal lining were not much altered; the small intestine was little changed, the large intestine, the lungs, heart, kidneys, and liver were in their normal state, the brain and vessels of the cerebral membranes were not very much filled with blood, nor was any exudation perceptible; the blood did not appear in any way changed, nor could the odour of alcohol be detected. Dilute alcoholic fluids produce no chemical change in the coats of the stomach, but, in the case of persons of temperate habits, chemical re-actions are to be apprehended from the immoderate use of a fluid containing 40 per cent. of brandy.

That the use of strong alcoholic fluids is followed by inflammation of the stomach, and that this has been detected in drunkards who died from the intemperate use of brandy, is a thing well known. When alcohol passes into the blood, it is not only conveyed thither by the veins, but it penetrates the tissues also, and in this way comes into contact with the vessels carrying blood; this happens the more easily in proportion as the alcohol is the more dilute. The phenomena which take place in consequence of the entrance of alcohol into the blood, and of its direct actions on the heart, brain, and spinal marrow, are at times not to be distinguished from those which follow by sympathy from the stomach; in the case of dilute alcoholic fluids, the former seem to predominate, whilst in the case of strong alcohol, the latter.

The chemical habitude of ether, with respect to organic matters, is at present of no importance in explaining its action. Ether produces no perceptible change on the albumen of the blood-serum; of all the constituents of the animal body it dissolves only the fat; epithelium, brought into contact with ether, is not changed; on the external skin ether produces cold by evaporation, and after some time it causes warmth and burning; on the contrary, when it is taken into the mouth it very soon occasions a sensation of burning, the epithelium affording a more rapid ingress to the ether than the epidermis; in wounds where the nerves are laid bare, a sensation of burning is very soon felt. A rabbit, into whose stomach a drachm of ether had been injected, seemed little excited by it, became very soon insensible, and fell on its side; the body became tympanitic, and, after fourteen minutes, death followed without spasms; on opening the body, the smell of the ether was very perceptible, the muscles contracted when mechanically irritated, the peristaltic motion of the intestine was very feeble; the stomach and small intestine were much inflamed, their contents did not appear changed, and there was only a little ether mixed with them, the cells of the gastric mucous membrane did not differ much from their normal form; in some places, where the mucous membrane was covered with blood, the epithelial cells were swollen to about six or eight times their natural size; the vascular membrane was much injected, the muscular and peritoneal coats were not changed, the small intestine was inflamed and contained much ether, its cylindrical cells were for the most part swollen. If, in cases where larger doses of ether were given, death followed very soon, the intestinal canal was not changed. Sometimes the animals die of suffocation amid the rapid evaporation of the ether. The ether accordingly occasions violent inflammation of the stomach and the intestinal canal; it penetrates the membranes, and is then conveyed into the blood-vessels. A chemical action on the tissues could not be perceived.

The structural change found in the intestinal canal, the swelling of the epithelial cells, and their removal is not a consequence of a chemical action, but is occasioned through the action of the organism; the general action of ether on the organism, partly from the stomach, and partly from its entrance into the blood, is therefore still unknown.—*(Ibid.)*

---

On the Anatomy and Diseases of the Urinary and Sexual Organs. By *G. J. Guthrie,* F.R.S. Third Edition.

Mr. Guthrie states, that time has confirmed the views he originally entertained of the Anatomy and Surgery of the Parts of which this work treats, while the experience which he has acquired, has enabled him to make the volume more worthy of the good opinion of the profession. It has been almost entirely reconstructed, and the directions for the treatment of each particular complaint are rendered so plain, that any one of even moderate capacity may understand and practise them. The work is now divided into two parts, of which this, which is the first, is devoted to the consideration of the Structure of the Bladder and of the Urethra, of the Formation of Spasmodic and Permanent Stricture, of the Symptoms of and Means of Cure of Stricture, of the Treatment of Impassable Stricture, of Suppression and Retention of Urine, and of Irritation of the Membranous and Prostatic parts of the Urethra.

The Second Part is to follow with the least possible delay; and will contain, the Chronic Complaints of the Prostate, the Diseases of the Bladder, the Treatment of Calculous Affections, and the various Modes of Operating for the Removal of a Stone from the Bladder.

The work is too well known to require any commendations on our part.

---

Principles of Forensic Medicine. By *Wm. A. Guy,* M.B. Cantab. Professor of Forensic Medicine, King's College. Part II. London, 1843.

The Second Part of this work has been just published. It commences with the subject of *Life Assurance.* The space devoted to the subject of Insanity, in this part, is very considerable, the author, as he himself states, being unwilling to pass over, or to treat too briefly, the grave questions which have recently occupied public attention. In his preliminary observations, on Unsoundness of Mind, he very properly lays it down, that a chief source of the difficulties connected with the subject of Insanity, may be traced to the prominence and importance formerly given, in works on the human mind, to one or two of its higher faculties. The reason and imagination were put so prominently forward, and the emotions and passions were made to play so subordinate a part, that soundness and unsoundness of mind came to be regarded as almost synonymous with a sound or erring reason; imagination had to bear all the blame of misleading the judgment, and delusion became the favourite test of Insanity. To the phrenologists our author justly ascribes the great merit of having directed attention to those faculties which are the real source of action—the emotions and passions; and to them he gives the praise of having originated the simplest and, by far, the most practical, theory of the human mind. There no longer exists any doubt respecting the soundness of the theory, that the mind is a compound of several faculties, capable of acting either alone or in combination, varying greatly in power in different persons, and in the same person at different times.

Once we admit the theory of the separate existence, and possible separate action, of the several faculties of the mind,—the reasoning faculties, the emotions or sentiments, and the passions,—and it is not more difficult to imagine a moral than an intellectual insanity; let us only allow that the several faculties, originally of different power in different persons, may combine in many different ways, and we have the materials of an almost infinite variety of character; the key to endless diversities of opinion, and the explanation of all that is most obscure in the motive and conduct of mankind. On the very important subject of moral mania our author is very happy. It is well known that, previous to the time of the humane Pinel, insanity was generally considered as either exclusively, or chiefly, a malady of the reasoning faculties. Influenced by the prevalent metaphysical doctrines of his time, according to which the existence of a few faculties were sufficient to explain all the phenomena of the sound and unsound mind, this great man found, to his great surprise, that there were many maniacs, who betrayed no lesion whatever of the understanding, but were under the dominion of instinctive and abstract fury, as if the affective faculties alone had sustained injury. The most practical observers now recognise the reality and importance of the distinction between intellectual and moral mania. In fact, according to Pritchard, moral generally precedes intellectual insanity. For excellent and valuable information on the proximate causes of Sudden Death—of death commencing in the HEART (*Syncope)*—in the HEAD (*asthenia*), and in the LUNGS (*asphyxia apnœa*), we beg leave to refer to the Chapter on Sudden Death, (p. 300.) In closing our notice of this part of Dr. Guy's work we have no hesitation in recommending it to the careful perusal of medical men, both students and practitioners. The compact form, and great condensation, which characterise it, cannot fail to procure it purchasers among those whose numerous avocations will not afford them time to peruse more voluminous works. The typographical elegance with which the work is executed, is in strict accordance with the well known taste of the publisher.

---

ELEMENTS OF CHEMISTRY, INCLUDING THE MOST RECENT DISCOVERIES AND APPLICATONS OF THE SCIENCE TO MEDICINE AND PHARMACY, AND TO THE ARTS. By *Robert Kane,* M.D. M.R.I.A. Professor of Natural Philosophy to the Royal Dublin Society; Professor of Chemistry to the Apothecaries' Hall of Ireland. Dublin, 1842; Hodges and Smith. London, Longman and Co.

BY some unaccountable over-sight we have, up to the present time, omitted to notice this work of Professor Kane. We assure the author the omission was wholly unintentional. His high and well-established character, as a Scientific and Practical Chemist, his numerous and ingenious discoveries in his favourite science, discoveries which have, at this early period of his life, acquired for him a more than European fame, are a sufficient guarantee for the merits of any chemical work coming from Dr. Kane. The present work is now so universally acknowledged, as the best text-book of Chemistry in the English Language, as to supersede the necessity of any remarks from us. If external reference, with respect to the great merits of Kane's Elements of Chemistry were necessary, we might point to the high character given of it by the greatest chemist of this or of any other country, we mean the distinguished and worthy successor of Sir Humphrey Davy, Faraday, who has declared Professor Kane's work the best Introduction to Chemistry that has as yet appeared, and has recommended its adoption in the Royal School of Woolwich. Among the many excellent qualities of this work, we were forcibly struck by the great simplicity of the author's style, which is such as to render even the most complicated parts of the science intelligible to the veriest tyro in chemistry—while to the more advanced student it cannot fail to recommend itself by the circumstance of its containing all the latest improvements and discoveries in Chemical Science. We perceive that our American friends are preparing a new edition.

---

Elements of Natural Philosophy; being an Experimental Introduction to the Study of the Physical Sciences. By *Golding Bird*, A.M. M.D. F.L.S. Assistant-Physician to Guy's Hospital, &c. London, Churchill. Pp. 479, 300 woodcuts. Second edition.

No one now thinks of raising the question, *cui bono*, regarding the utility of any branch of knowledge connected, however indirectly, with our profession. Independently of the actual importance of the several sciences included under the comprehensive word "physics," to all, especially to the student of medicine, much indirect good arises from their study, in the discipline of the mind produced by an acquaintance with the inductive sciences. It were well for the medical profession generally, if its members took a lesson from the mode in which successful researches in physics have been ever carried on, for by accustoming themselves to reason from the known to the unknown, they would be less likely to rush too hastily to conclusions when the premises are either obscure, or scarcely thought worth examining.

The rapid sale of the first impression of this work is a sufficient proof of its merit having been appreciated, and Dr. Golding Bird has availed himself of the opportunity thus afforded of much improving and enlarging his book, by the addition of an account of many of the numerous discoveries in science which the last four years have produced. In pronouncing this edition, as the most useful and complete manual of physics before the public, we feel that we are doing its author but bare justice. It is, in fact, the only work we possess which the student can use as a text-book in commencing the study of his profession, especially in preparing himself for his matriculation examination at the Metropolitan University.

The numerous woodcuts which appear in almost every page, afford no small assistance to the student in comprehending the *penetralia* of the subjects treated on, and will greatly diminish his labours. We again cordially recommend Dr. Golding Bird's volume, as the most comprehensive and valuable manual of physics we possess.

---

Semeiotique des Urines, ou Traite des Alterations de l' Urine dans les Maladies; suivie d' un Traite de la Maladie de Bright aux divers Ages de la Vie. Par *Alfred Becquerel*, D.M. &c. &c. Paris, 1841.

Semeiology of the Urine; or a Treatise on the Changes of the Urine in Diseases; followed by a Treatise on Bright's Disease at the different Ages of Life. By Dr. *Becquerel*, &c. &c,

This work has been divided into four parts; the first part treats of *the Chemical and Physical Properties of the Urine.* The Ancients made a distinction between the urine passed at different periods of the day: thus they admitted three species of urine:—1. *Urina potûs.* This name they gave to the urine voided after swallowing a certain quantity of liquid, whether this liquid was drunk during meals, or in the intervals between meals: this urine depends on the quantity of liquid drunk; it is generally clearer, more limpid, and less dense than the urine passed under other circumstances. 2. *Urina digestionis.*—This is the urine passed two or three hours after meals, and when the digestion is partly, or entirely completed. This urine is influenced by the quantity and nature of the aliment introduced into the system. It is denser, more highly-coloured, and less abundant than the preceding. 3. *Urina sanguinis,* or the urine of the morning; which may be considered as the pure product of the renal secretion. This species of urine is connected with the composition of the blood, and may be considered as influenced in the least possible degree by the liquids and aliments introduced into the system the preceding day. This urine is deeper, more dense, and more acid than the two preceding species. The distinction of these three species of urine is of great importance, and it is not improbable that the difference found between the analyses of the urine as given by different chemists should be attributed to the circumstance of their not having collected the urine for examination at the same period of the day. Thus the quantity of urea and of uric acid, given by Berzelius, is evidently greater than it would have been, had he taken the mean of the three species of urine. In diseases capable of occasioning any changes in the composition of the urine, it may be laid down generally that, in the great majority of cases, this distinction no longer exists, except only those cases where a great quantity of liquid has been taken into the system in the space of twenty-four hours. In these diseases the urine, except in the exceptional cases, is similar to itself, at whatever part of the day it may be collected. Still the urine voided in the morning was that selected by the author for experimentation, because that urine bears a nearer relation than any other to the composition of the blood, and because, when collected under the same circumstances, it is always like to itself. These same reasons have induced most physicians, when studying the properties of the urine, to select the morning urine. There is a crowd of questions, however, which the physician may put to himself and which the examination of the morning urine only will not enable him to answer. Thus, for instance, the urine of an individual who labours under intense febrile re-action is denser, more acid, and more highly-coloured than in a healthy individual, and further, it presents frequently a copious sediment of uric acid combined with the colouring matter. Such urine contains, then, more uric acid, and more animal matters than in the healthy state. Now this increase may not be real, it may be only apparent, and relative. Who can say with certainty that it may not depend on the diminution of the quantity of water, and the greater concentration of the different solid principles? Another method of proceeding has been suggested by M. Chossat, and adopted by M. Lecanu; it consists in collecting all the urine passed in the twenty-four hours, mixing them together and analysing the mixture. This method has been adopted by the author in a great number of his experiments; it is not, however, he acknowledges, entirely free from error, and is attended with difficulties.

In Chap. II. M. Becquerel gives the *Composition* of the Urine in the normal state. He begins by laying it down as a general principle, that the urine passed in the space of twenty-four hours, or that passed in the morning by the same individual, is never absolutely and identically of the same composition on any two or three consecutive days, in a word, that the mean quantity of each chemical element is never absolutely the same; it varies within limits which in general are not considerable, but which, however, may become so. Thus, for instance, M. Lecanu found that, in man, the urea secreted in twenty-four hours may vary between 22·446 grains and 9·119 grains, and in women between 17·983 grains and 9·881 grains; it is the mean of these oscillations that are given in the Table exhibiting the composition of the urine in the normal state. When the quantity of such or such a principle obtained by the analysis of urine voided in a state of disease is to be compared to this, it will be necessary to examine whether this quantity differs little or much from the normal mean: if it differ but little, the conclusion will be that it is not beyond the healthy limits.

TABLE, *exhibiting the Mean Composition of the Urine in the Male and Female, in the Normal State, and the Mean deduced from their addition. This Table is constructed from the results of the analyses of* 8 *specimens of healthy urine,* 4 *of Men and* 4 *of Women.*

| Chemical Elements contained in the Urine. | Males. | | Females. | | General Mean. | |
|---|---|---|---|---|---|---|
| | Urine of 24 hours. | Composition in 1000 parts. | Urine of 24 hours. | Composition in 1000 parts. | Urine of 24 hours. | Composition in 1000 parts. |
| Quantities of Urine.. .. .. .. .. .. | 1267·3 | 1000 | 1371·7 | 1000 | 1319·8 | 1000 |
| Density .. .. .. .. .. .. .. | 1018·900 | | 1015·120 | | 1017·010 | |
| Water .. .. .. .. .. .. .. | 1227·779 | 968·815 | 1337·489 | 975.052 | 1282·634 | 971.935 |
| Other matters than water afforded by direct evaporation.. | 39·521 | 31·185 | 34·211 | 24.948 | 36·866 | 28.066 |
| Urea .. .. .. .. .. .. .. .. | 17·537 | 13·838 | 15·582 | 10.366 | 16·555 | 12·102 |
| Uric acid .. .. .. .. .. .. .. | 0·495 | 0·391 | 0.557 | 0.406 | 0·526 | 0·398 |
| Salts fixed and not decomposable at a red heat .. .. { Chlorides, Phosphates, Sulphates } { Lime .., Soda .., Potass .., Magnesia } | 9·751 | 7·695 | 8·426 | 6.143 | * 9.089 | ** 6·919 |
| Organic matters which cannot be isolated, and estimated separately .. .. .. { Lactic Acid .. .. .. .., Lactate of Ammonia .. .., Colouring matters . .., Extractive matters .. .., Hydrochlorate of Ammonia .. } | 11·738 | 9·261 | 9.655 | 8.033 | 10.696 | 8.647 |

*Composition of the Fixed Salts in the Urine voided in* 24 *hours, aud in* 1000 *parts.*

* Urine of 24 hours:—

| | |
|---|---|
| Sum . . . . . . . . . . . . . . . | 9·089 |
| Chlorine . . . . . . . . . . . . . . | 0·659 |
| Sulphuric acid . . . . . . . . . . . . | 1·123 |
| Phosphoric acid . . . . . . . . . . . | 0·417 |
| Potass . . . . . . . . . . . . . . | 1·708 |
| Alkaline and Earthy bases . . { Soda, Lime, Magnesia } . | 5·181 |

** Composition in 1,000 parts:—

| | |
|---|---|
| Sum . . . . . . . . . . . . . . . | 6·919 |
| Chlorine . . . . . . . . . . . . . . | 0.502 |
| Sulphuric acid . . . . . . . . . . . . | 0.855 |
| Phosphoric acid . . . . . . . . . . . | 0·317 |
| Potass . . . . . . . . . . . . . . | 1·300 |
| Alkaline and Earthy bases . . { Soda, Lime, Magnesia }. | 3·944 |

The quantity of water voided in the space of 24 hours, may be represented by the following means, or rather by variations or oscillations around these means:—

| | | |
|---|---|---|
| In males . . . . . . | 1227·779 | grains. |
| In females . . . . | 1337·489 | grains. |
| General mean . . . | 1282·634 | grains. |

Whenever the numbers are found to vary from 800 to 1,500 grains of urine passed in 24 hours, these numbers are not to be considered as without the physiological limits—but to admit a morbid alteration in the quantity, its variations must be below 800 grains, and above 1,500 grains. The quantity of water may increase, and also may be diminished. One physiological circumstance and three great pathological causes may increase this quantity. This great physiological cause is the swallowing of a great quantity of liquid, whilst the three pathological causes are the following:—1, Polydypsia; 2, Diabetes; 3, an attack of hysteria, or any nervous affection.

The case of a diminution in the quantity is much less frequent than its increase. The causes of this diminution are the following:—1. Fever, and, therefore, all circumstances capable of causing febrile re-action, more especially acute and chronic inflammation. 2. Diseases of the heart and liver. 3. Diseases of whatsoever kind, capable of exciting a general functional disturbance of the system. 4. Profuse sweats. 5. The near approach of death. The number expressing the normal quantity of water contained in 1000 grains of urine oscillates around the number 971·934 grains, sometimes more, sometimes less—this number may increase or diminish. The cases wherein the quantity of water increases with respect to the matters it holds in solution, are chlorosis, and the various anæmic diseases.

*Of the Solid Principles held in Solution in Water.*

The Sum of the solid principles held in solution in water is given by direct evaporation. The means representing these in the normal state are the following:—

| | *Water.* | *Solid Principles secreted in* 24 *hours.* |
|---|---|---|
| Males . . . . . . | 1227.779 | 39·521. |
| Females . . . . . . | 1337·489 | 34·211. |
| General mean . . . | 1282·634 | 36·866. |

These means are not the same in the male and female; nor are they invariably the same even in the same individual. In the male the variations may be between 36 and 41—in the female, between 32 and 36. The mean oscillations in the two sexes may be between 32 and 41.

The quantity of solid principles imparts variable qualities to the urine; according as they are dissolved in a greater or less quantity of water. The amount of these matters held in solution may vary under certain physiological or pathological influences. 1st. The causes which increase them are the following:—1, a rich, abundant, azotised diet; 2, the introduction into the system of an abnormal quantity of water; 3, polydypsia; 4, nervous affections, and more especially hysterical paroxysms; 5, diabetes. The amount of the solid principles held in solution in the urine, is very frequently diminished in disease; this is a much more common occurrence than the preceding. 1st. Under the influence of fever, acute inflammation, functional disturbances, diseases of the heart or lungs, diseases of the liver, the quantity of solid matters secreted in 24 hours is diminished. 2nd. Under the influence of these same conditions, if there also exists exhaustion, debility, or anæmia, the amount of the solid principles held in solution is considerably diminished. 3rd. Under the influence of entirely opposite states, the amount of the matters held in solution is observed to diminish.

These causes are—1. Chlorosis. 2. Anemia. 3. Debility, produced by entirely different causes, losses of blood, purulent discharges, &c. &c. 4. Exhaustion occasioned by chronic diseases.

*Urea.*—In the healthy state the mean of the quantity of urea contained in 1000 grains of urine oscillates around the following numbers. Urine having the following mean densities:—

| | | |
|---|---|---|
| In males, | D. 1018·900, urea | 13·838 |
| In females, | D. 1015·120, id. | 10·366 |
| General mean, | D. 1017·010, id. | 12·102 |

As well as the quantity, so also the quality, of the urea is liable to change; every time the urine becomes alcaline, and this frequently occurs, whether this takes place within the body, or results from the decomposition of the urine out of the body, this change is the consequence of changes in the urea.

*Uric Acid.*—This acid, though existing in the urine in small quantity, is, however, one of its most essential elements, and one which varies most in diseases. It has been made a subject of enquiry whether this uric acid exists perfectly free in the urine, or is combined with a base, and in the state of an acid urate, and more especially an acid urate of ammonia. The uric acid is not in the state of perfect freedom; for the urine, whether spontaneously, or under the influence of a re-agent, scarcely ever throws down crystallised uric acid. On the other hand, these sediments, when examined with the microscope, are found to consist of a substance which presents itself under the appearance of a very fine, amorphous powder. According to Quevenne, with whom our author agrees, this powder is neither more nor less than uric acid, which has become amorphous by reason of its combination or mixture with animal matters, as also with the colouring matter of the urine.

With respect to the usual physiological limits of uric acid, our author gives the following analysis:—

1. *Quantity of Uric Acid, compared to that of the other Elements, the Urine being* 1000.

| | Density. | Uric Acid. |
|---|---|---|
| Males | 1018·900 | 0·391 |
| Females | 1015·170 | 0.406 |
| General mean | 1017·010 | 0·398 |

2. *Quantity of Uric Acid secreted in* 24 *hours.*

| | Quantity of Urine. | Density | Uric Acid. |
|---|---|---|---|
| Males | 1267·3 | 1018·900 | 0·495 |
| Females | 1371·7 | 1015·120 | 0.557 |
| General mean | 1319·5 | 1017·017 | 0·526 |

From these tables we may conclude that, in 1000 parts of urine, the physiological mean varies between 0·3 and 0·5,—and in the quantity secreted in 24 hours, between 0.4 and 0.6.

Want of space prevents us from continuing this analysis.

### Cataract and its Treatment, comprising an Easy Mode of Dividing the Cornea for its Extraction, &c. By *John Scott*, Esq.

Mr. Scott states that, he has long considered the chief danger attending the extraction of cataract has arisen from the force that is necessary to transfix the cornea with the instruments commonly employed for that purpose; and that spasm of the recti muscles, induced by that force, compressing the iris between the hard lens and the side of the knife, and occasioning inflammation of that membrane, has been the most frequent cause of an unfavourable result of the operation. To obviate these inconveniences, Mr. Scott has invented a new knife, the utility of which, he states, he has tested in a great number of cases.

The cornea-knives usually employed, not only increase in thickness and in width from point to heel, to fill up the aperture they make in the cornea, as they traverse the anterior chamber, and thus prevent the escape of the aqueous humour, but their width is also equal to the radius of the cornea, so as to make a section of that size in the membrane; and this is done by thrusting this wedge-shaped instrument through the cornea. Now the force necessarily employed for this purpose tends to turn the eye inwards, obscuring from view the inner margin of the cornea, and rendering it difficult to puncture the cornea close to the sclerotic edge, and frequently causing the section to be too small for the escape of the cataract. If this inversion of the eye is opposed by pressure on the nasal surface of the globe, the aqueous humour is apt to escape, and the iris is in danger of being wounded; or even if the iris is uninjured, spasm of the muscles is occasionally produced, endangering the escape of the vitreous humour.

"Sometimes the spasm thus induced will not subside after the extraction of the cataract, and then the iris may be pressed forward and prevent the closing of the flap of the cornea; under these circumstances, it is necessary to puncture the hyaloid membrane and allow a small quantity of the vitreous humour to escape, which will relieve the spasm, unless it should subside spontaneously, after waiting a reasonable time for the purpose." Sometimes, when the vitreous humour escapes, a portion of the hyaloid membrane may be left protruding through the section of the cornea, when it must be returned by the silver end of the curette.

A needle can be introduced into the anterior chamber without difficulty, and retained there for some time without the escape of the aqueous humour; hence it occurred to Mr. Scott, that if a knife could be constructed that might be introduced into the eye with as little force as is necessary for the introduction of the needle, and of such shape as would complete the section of the cornea without danger of wounding the iris, the difficulties and the risk attending the operation would be most materially lessened. In the usual way of operating, the knife cuts its way *into* the cornea, which requires considerable force, but Mr. Scott's knife is introduced into the anterior chamber without any further division of the cornea than is necessary for the purpose of its introduction, the section of the membrane not being commenced until both sides of the cornea have been punctured; and the knife is of such a shape, and is then so situated that there is little danger of the iris falling forward before its edge.

The following is the description of the knife employed by Mr. Scott. "The back of the knife describes the sixth part of the circumference of a circle, the radius of which is ten lines. The chord of the arc formed by the back of the knife, is, of course, also ten lines in length, being equal to the radius of that circle; it is therefore greater by four lines than the diameter of the cornea, and the blade is consequently quite long enough to complete the section of that membrane without difficulty. The knife is two lines in width at the heel, whence it gradually tapers to the point; it also increases uniformly in thickness as well as in width from point to heel, so as to occupy completely the aperture it makes in the cornea, for the purpose of preventing the escape of the aqueous humour."

In making the upper section of the cornea with this knife, it is to be held in the usual manner, the cornea punctured on the temporal side, and the point of the knife carried across to the nasal side, till it reaches the nasal canthus of the orbit; its cutting edge will then be so far beyond the pupillary margin of the iris that it cannot be readily divided in completing the section of the cornea. The point of the knife is then to be carried upwards, the handle being slightly inclined in the opposite direction. The section of the cornea on its nasal side will now be complete, a small portion of the upper and outer part only remaining to be divided; and this is readily done on the withdrawing of the instrument.

If the case go on favourably after the operation, the patient will scarcely complain of any pain; there will be some soreness and uneasiness, which will be relieved by a discharge of tears. It is necessary to cleanse the margins of the lids with a rag and warm water occasionally, as they are liable to become agglutinated by a secretion of mucus; bathing the lids frequently with warm water will often alleviate any pain that comes on after the operation. Should the pain be very severe it may be necessary to give a full dose of opium.

It is a very common practice to take blood from the arm both before, and in the evening after the operation, for the purpose of preventing any inflammation; Mr. Scott, however, asserts that in fifty cases of extraction performed at the Ophthalmic Hospital, taken in succession, he has not had occasion in a single instance to abstract blood either before or after the operation. It is evident that the indiscriminate abstraction of blood from the feeble and aged persons who are so frequently the subjects of cataract, instead of obviating the occurrence of inflammation, will rather tend, by reducing the patient's powers, to prevent the closing of the section by the first intention, and thus induce the suppurative process, which will be very liable to be attended with violent inflammation of an asthenic character, that it will be very difficult, if not impossible, to control in this reduced state of the system.

The description of the various forms of cataract is plainly and sensibly written.

---

### Researches on the Decomposition and Disintegration of Phosphatic Vesical Calculi, and on the Introduction of Chemical Decomponents into the Living Bladder. By *S. Elliott Hoskins*, M.D.

It has long been a desideratum to discover some agent sufficiently powerful to act directly on the stone while in the bladder, and at the same time so mild as to produce no irritation of the tissues of which the bladder is composed. Hitherto it appears to have been the object to act on calculi by *single* elective affinity only; that is, to dissolve the base before it is disengaged from its associated acid. Dr. Hoskins, however, attempts to arrive at the proposed end by bringing into play *complex* affinity; that is, to find an agent, the base of which shall be so attractive of the acid of the calculus, as to withdraw it from its allegiance; whilst the acid of the agent unites with the basic ingredients of the calculus, to form with them salts of easy solution.

The facility with which salts of lead decompose the phosphates seemed to point them out as agents well-fitted for the proposed purpose. On immersing, however, fragments of phosphatic calculi in solutions of neutral acetate of lead, no chemical action was discernible.

According to Dr. Prout, fluids containing malic acid possess peculiar powers in arresting the deposition of the phosphates. From this circumstance, and also on account of the solubility of the salts which malic acid forms with the bases of these calculi, viz. magnesia, lime, &c., Dr. Hoskins was led to make trial of a malate or super-malate of lead. To obtain this, a watery solution of neutral acetate of lead was added to cider-vinegar, or *cidre-aigre,* till precipitation no longer took place. The liquid, on being filtered, was clear, devoid of acidity or acrimony, and possessed a pleasant sweetness, unmingled with any metallic astringency.

On immersing fragments of phosphatic calculi in the liquid thus formed, rapid chemical action ensued, visible to the naked eye. On suspending a fragment of fusible calculus by means of horse-hair, in a test-glass, containing the fluid, it became at once involved in a white cloud, from which a continuous stream of precipitate gravitated to the bottom of the glass; this precipitate was chiefly phosphate of lead. After the lapse of half an hour, the calculus was found to have lost weight. This was an important fact, without which it might have been supposed that decomposition of the solution alone had given rise to the precipitate. The liquid thus formed from the *cidre-aigre* was, however, objectionable in a practical as well as in a scientific point of view, on account of its colour, odour, and indeterminate strength. It became therefore desirable to attempt the formation of some definite salt, of analogous composition, which, by solution in water, should form a decomposing agent, of a strength which might at all times be depended on, capable also of being varied according to circumstances.

After many experiments, the salt selected was the nitro-saccharate of lead, formed by dissolving a portion of pulverized saccharate of lead in a sufficient quantity of cold dilute nitric acid. The solution, on being filtered and gradually evaporated, yields a quantity of perfectly transparent, amber-coloured crystals, in the form of regular hexagonal plates or prisms. One grain of this salt, moistened with five drops of pure saccharic acid, and dissolved in a fluid ounce of distilled water, formed a blank liquid without any astringency, although it possessed slight acid re-action. It acted with rapidity on various specimens of phosphatic calculi, forming around each, at the moment of immersion, a dense nebula, from which a white precipitate subsided. Chemically speaking, this was the most active agent yet experimented with, whilst its sensible character was so mild, as to be tolerated with perfect impunity by the urethral and conjunctival membranes.

Having ascertained the effects of these salts of lead on phosphatic calculi, out of the body, the next point to be determined was the effect likely to be produced by the introduction of their solutions into the living bladder. Some fear might perhaps be entertained with regard to the production of colica pictonum;—this is to be guarded against by adding to the salt of lead employed, a small quantity of its own proper acid, or a few drops of pure acetic acid, *previous* to the addition of water. In a chemical, as well as a therapeutical point of view, this is essential: 1st, as it secures the perfect solution of the salt, and its consequent activity as a decomponent; 2nd, the superaddition of acid secures against the formation of any of the deleterious carbonate.

In order, however, to be perfectly satisfied as to the comparatively innocuous character of these salts, Dr. Hoskins undertook a number of experiments with them on sheep, introducing the fluids into the bladder daily, for several weeks consecutively, and having the animals killed at different periods during the investigation. In none of the sheep experimented on, were untoward symptoms excited, either general or local. Experiments also of a personal nature were resorted to, and contributed to the conviction that no evil could accrue from the continued introduction of saturnine solutions into the bladder.

Three cases are related in which these injections were employed, the bladder being in an irritable state from the presence of calculi; these cases, though very unfavourable for testing the lithontriptic powers of the solution, afforded evidence of its being neither irritating nor injurious, when introduced with proper restrictions into the bladder.

